# Towards TB elimination: how are macro-level factors perceived and addressed in policy initiatives in a high burden country?

**DOI:** 10.1186/s12992-020-00657-1

**Published:** 2021-01-11

**Authors:** Prince A. Adu, Jerry M. Spiegel, Annalee Yassi

**Affiliations:** 1 School of Population and Public Health, University of British Columbia, 2206 E Mall, Vancouver, BC V6T 1ZE Canada; 2 British Columbia Centre for Disease Control, 655 W 12th Ave, Vancouver, BC V5Z 4R4 Canada

**Keywords:** Tuberculosis, Structural determinants, South Africa, Perception, Health system

## Abstract

**Background:**

Notwithstanding extensive general discussion of the effects of upstream forces on health, there has been limited empirical examination, let alone systematic evidence documenting policy responses to such pathways in the area of tuberculosis (TB) management and control. Our study aimed to gain insight into how macro level drivers of TB are perceived by key stakeholders involved in TB management and control in a high-endemic country, and to assess how such concerns are being addressed in policy initiatives in this setting. South Africa was chosen for this case study due to our team’s long-standing collaborations there, its very high burden of TB, and its introduction of a strategic plan to combat this disease.

**Method:**

Semi-structured interviews were conducted with 20 key informants who were purposively selected for their knowledge and expertise of TB in South Africa. South Africa’s *National Strategic Plan for HIV, TB and STIs 2017–2022* was then reviewed to examine how identified themes from the interviews were reflected in this policy document.

**Results:**

A history of colonization, the migrant labour system, economic inequality, poor shelter, health system challenges including TB governance, the HIV epidemic, and pertinent socio-cultural factors were all perceived to be major drivers of the epidemic. Although South Africa’s current *National Strategic Plan* makes a firm discursive commitment to addressing the structural or macro-level drivers of TB, our analysis revealed that this commitment was not clearly reflected in projected budgetary allocations.

**Conclusion:**

As in many other high burden settings, macro-level drivers of TB are widely recognized. Nonetheless, while micro-level (biomedical and clinical) measures, such as improving diagnostic procedures and investment in more efficacious drugs, are being (and well should be) implemented, our findings showed that macro-level drivers of TB are underrepresented in budgeting allocations for initiatives to combat this disease. Although it could be argued that structural drivers that undermine health-promoting actions are beyond the purview of the health sector itself, we argue that strategic plans to combat TB in high burden settings need more attention to directly considering such drivers to prompt the necessary changes and reduce the burden of this and other such diseases.

## Background

Tuberculosis (TB) is a preventable and curable condition that remains among the top ten causes of death worldwide, killing about 1.8 million people each year [1]. It is widely agreed that TB rates reflect the economic and socio-cultural conditions within a country, as well as other macro-level or structural determinants of health [2–4]. As a disease of poverty, TB is driven by structural or upstream forces and disproportionally affects the economically disadvantaged and marginalized in society, with highly uneven distribution globally. Throughout this paper we use the terms “upstream”, “systemic” or “macro-level” factors to refer to processes that modify or affect traditional proximate risk factors of TB disease. Notwithstanding the debates and nuances each of these terms hold, these terms are used interchangeably throughout this article. Proximate factors are those circumstances related to direct exposure to infectious droplets (including what are sometimes referred to as “meso-level” factors such as hospital infection control measures) and/or individual level factors (“micro-level”) that impair the host’s defense against TB (such as human immunodeficiency virus [HI]V, malnutrition, tobacco smoke, alcohol, silicosis, diabetes and others) [2].

It is increasingly well-established that upstream economic forces reduce fiscal space for national governments to invest in health and social programs, thereby limiting the recruitment and retention of adequate numbers of well-trained medical and nursing personnel needed to address the various challenges in health systems. Such policies also limit investing in social protection in general [5]. With respect to TB itself, a recent modelling analysis of the 22 high-burden TB countries, performed by the lead author, conducting his doctoral research on this topic at the time, added to the evidence that macro-level factors have a significant impact on TB incidence [6]. This added further evidence to previous predictive modelling that demonstrated that global TB incidence would be reduced by 84·3% if poverty were eliminated; and implementation of social protection measures alone would reduce tuberculosis incidence by 76·1% [7]. Various recent studies have indeed shown that cash transfers to patients with TB might improve treatment outcomes [8–11].

As a social disease, experts and researchers largely agree on TB’s systemic underpinnings. Karim and colleagues argue that the social, economic and environmental conditions that were created in South Africa as a result of the Apartheid system have favoured the growth and transmission of TB [12]. Weyer and colleagues identified the legacy of neglect of the disease, its poor management and the fragmented health services as three main drivers of the TB epidemic in this country [13]. Other barriers to effective TB control in South Africa have been identified as the HIV epidemic, poor socioeconomic conditions and the shortage of human resources for health [12].

Notwithstanding these recent studies, the evidence base regarding government policy responses to known local socioeconomic factors hindering the treatment of TB, let alone to address well-known macro-level drivers of TB, is still very limited. A recent article detailing evidence-informed policy-making from a South African TB think tank [14] did not explicitly consider these factors.

Globally, the WHO End TB Strategy aims to reduce the mortality and morbidity of TB by 90 to 95% respectively [15, 16]. In this context, the South African *National Strategic Plan (NSP) for Human Immunodeficiency Virus (HIV), TB and Sexually Transmitted Infections (STIs) 2017–2022* calls for reducing TB incidence (including HIV+ TB) from 450, 000 to 315, 000 by the year 2022 [17]. To meet these ambitious goals, the macro level conditions that enable TB to thrive need to be effectively addressed.

We therefore sought to investigate perceptions of macro-level drivers of TB by decision-makers and TB experts in high burden countries, and to ascertain how these perceived macro-level priorities are being addressed in policy and budgetary allocations to reduce the TB disease burden. The rationale is that discordance between stakeholders’ perspectives and policy directions may point out gaps and suggest the need for further policy considerations.

Given the limited empirical data on policy responses to such drivers in the health sector generally, we chose to conduct a case study in South Africa. South Africa was chosen for this case study due to its very high burden of TB, its introduction of an *NSP* [17], and our team’s long-standing collaborations that have emphasized prevention and control of TB among health workers [18–23] needed to facilitate access to TB experts and decision-makers.

In 2016, South Africa reported an incidence of 781 cases per 100,000 population, and mortality rate of 101 per 100,000 [1], making it one of the highest TB burden countries. Historically, the South African TB programme has mainly focused on traditional curative approaches, with particular attention to investment in more modern rapid diagnostic tests for drug-susceptible and drug-resistant TB, such as the GeneXpert test [24]. Efforts have also been geared towards the development of new treatments for multi-drug resistant (MDR) and extremely drug resistant (XDR) TB. Other initiatives to reduce the burden of TB include primary healthcare re-engineering to increase the accessibility and quality of healthcare services, and the adoption of efficient community-based treatment models [25]. Although these approaches have had some success in reducing TB mortality in South Africa, this reduction has been slower than expected [2, 4]. As such, a re-examination of current approaches to tackle the epidemic in this country was undertaken to better comprehend how drivers of TB are being understood and addressed.

## Methods

### Design

Twenty-three [23] potential key informants (KI) were purposively identified based on their knowledge, expertise and role in developing and/or implementing TB-related policies in the South African context. Letters were sent to potential KIs to request their participation in the study. Of the 23 potential participants, 20 agreed to participate. Semi-structured interviews were conducted with the KIs; 15 interviews were conducted in person, four by telephone and one by Skype. Interview questions were provided to the participants in advance, and interviews lasted between 40 and 90 min. Questionnaire items were aimed at eliciting the KIs’ perception of the structural forces that drive TB infection in South Africa’s general population. To best elicit honest perceptions and maintain anonymity of the KIs, all interviews were conducted by the lead author, without direct participation of South African colleagues.

The key informants method [26] has been deemed to be particularly appropriate for such an inquiry where an established evidence base is not recognized. This method also provides a way to consider the existence of potential biases or omissions in perceptions by those who may be in a position to contribute to such decisions [27].

### Participants

The 20 KIs were recruited from five major categories of expertise: 1) provincial and national government officials with a TB-related portfolio (designated as “G” in the quotes); 2) academic experts in the field of public health or occupational health (“A”); 3) TB Advocacy Group/Non-Governmental organizations (“N”); 4) TB hospital head (“H”); and 5) legislator (“L”). These are professionals who have a good understanding of the epidemiology of TB and its resulting consequences. Table 1 contains more information of the KIs.

**Table 1:** Key informants’ refence information (identifying information has been removed to protect KIs’ anonymity)

Reference	KI Category	Designation	Mode of Interview
G1	Provincial and national government authorities	Senior level manager in charge of occupational hygiene in a provincial health department	In person
G2		Mid-level manager in charge of occupational hygiene in a provincial health department	In person
G3		Mid-level manager in charge of occupational hygiene in a provincial health department	In person
G4		Mid-level manager in charge of occupational hygiene in a provincial health department	In person
G5		Senior level manager in charge wellness program in a provincial health department	In person
G6		Senior manager in charge of TB, national department of health	In person
G7		Senior manager in charge of hygiene and health, national labour department	In person
G8		Senior manager, national department of public service	In person
G9		Senior manager, national institute of occupational health	In person
G10		Occupational medicine specialist of a national institute	In person
A1	Academic experts	Professor emeritus of occupational medicine	Skype
A2		Professor in occupational health in a public university	In person
A3		Professor in a public university	In person
A4		Professor in a public university	In person
N1	TB Advocacy Group/Non-Governmental organizations (NGOs)	Survivor of MDR TB and TB advocate/former HCW	Telephone
N2		Survivor of MDR TB and TB advocate/HCW	Telephone
N3		NGO official	
N4		Professional group official	
H1	Hospital heads	Head of a large TB referral hospital	In person
L1	Health Legislator/Politician	Legislator	Telephone

### Data analysis

All interviews were audio-recorded, transcribed by hand and exported into NVivo 11 (*NVivo qualitative data analysis Software*, 2015) for analysis, following standard informed consent procedures to ensure confidentiality and protection of the identity of the respondents. Each transcript was read several times and a priori coding was conducted on the transcripts using predetermined themes that were developed from the objectives of the study. Recognizing the limitations of a priori codes, emergent codes were also identified within each interview and aggregated into categories. To ensure credibility, inter-coder agreement and internal validity, a secondary data analyst reviewed the transcripts to seek agreement on coding.

After the analysis of the interviews was completed, the current South African NSP for HIV, TB and STIs was then analyzed to determine the extent to which the themes identified by the KIs were reflected in the focus areas and goals of the NSP. For the purposes of this study, only sections of the NSP that dealt with TB were analyzed.

## Results

### Interview themes

The perceptions of KIs emphasized the following themes: colonization, the political and economic system; the mines and migrant labor system; racial and economic inequality, poverty and malnutrition; infrastructural challenges; health system challenges, such as poor infection control, treatment-related factors and resource allocation; the HIV epidemic, and socio-cultural factors. The text is presented with quotes from the KIs to highlight the main points.

#### Colonization, political and economic system

Distinct historical events and the current socio-economic environment were cited as instrumental in driving the TB epidemic in South Africa. Four KIs identified European miners as the source of the epidemic. According to their account, native Black Africans lacked immunity to TB because they had never been previously exposed to the disease. A KI further blamed the epidemic on the impact of colonialism:

*“let me tell you what the major contributor of TB in South Africa is; TB is foreign in Southern Africa. It is from the northern hemisphere. South Africa had the highest number of White people and therefore the highest dose of TB.*” (N4).

This point was somewhat supported by the situation in the Western Cape where a relatively well-off province with a comparatively better health system has ironically one of the highest TB disease burdens in the country. The KI who made this argument explained:

*“The Western Cape has one of the highest rates of TB in South Africa, historically and it is because people moved from Europe when TB was not curable [and] came to South Africa. Western Cape has the second highest GDP after Gauteng province but the rates there [Western Cape] are highest, and I believe it is because of the high number of people who went there 200 years ago.”* (N4).

Some KIs asserted that the political system of South Africa, which, under the apartheid regime had formally and forcibly segregated Black people into poor living conditions, made it conducive for TB to thrive and resulted in increased TB transmission, especially in the Black communities.

### The mines and migrant labour system

Several KIs noted that migratory labour patterns remained a significant driver of the TB epidemic in South Africa. They explained that the migrant labour system employed since the late nineteenth century in the prominent mining sector, where miners go back and forth between their communities and mining areas, was (and to some extent still is) a major contributor to the spread of TB. Internal and external migration was cited as a contributing factor, and the direction of impact for spreading TB was noted to be multidirectional. One KI observed that:

*“We are known as a country that exports HIV and TB to labour serving countries like Swaziland, Mozambique, Lesotho and to some extent, Zambia and others because mine workers come to SA as migrant workers, get TB in the mines, go home to their wives and families.”* (G8).

KIs also expressed that the mining sector requires their workers to congregate in stuffy settings where there is little ventilation, thus exposing miners to infectious diseases such as silicosis and TB.

### Racial/economic inequality, poverty & malnutrition

Racial and socio-economic inequality was cited as a driving factor in the TB epidemic in South Africa. A government official noted:

*“[The health system] is deeply an unequal one. There is de-prioritization particularly of public health services divided along racial lines. If you look at the percentage of GDP expenditure on health as a whole, it is about 8.5 percent, but 4.4 percent is in the private sector and only 4.1 percent in the public sector.”* (L1).

The structural inequality created by the apartheid system, and which continues to exist in the country, was further emphasized by a KI as follows:

*“Socioeconomic issues like poverty, unemployment, overcrowding, and poor housing are the traditional structural drivers which have been there because of Apartheid – and which have now been perpetuated in the post-Apartheid settings. You have traditional structural inequalities that have been engineered because of the previous Apartheid system which has been perpetuated in the new democracy.”* (G8).

Almost every KI (17 out of the 20) noted that TB is driven by poverty, drawing attention to the fact that TB is often referred to as a disease of the poor. Poverty leads to poor nutrition, which reduces the immune response to infections including TB.

An official also explained that:

*“People in poverty [acquire TB] in the sense that they would not have the right nutrition status that would prevent them from getting diseases or the immune system they require, or they would find themselves in [an] environment that do not protect them from respiratory conditions.”* (A4).

*“The social justice aspect of TB plays a big role and this needs to be looked it”* (G3).

### Density, shelter, infrastructure

Poor shelter compels people to share spaces with poor ventilation. Overcrowded areas prevent free air circulation and increase TB transmission, constituting what most KIs expressed to be one of the most important environmental drivers of TB. KIs also indicated that the government had an important role to play in addressing this. A KI explained this perspective in the following quote:

*“In terms of environmental factors, I don’t think our government [agencies] talk to each other or talk about it. In terms of housing, we are building all these RDP [Reconstruction and Development Programme] houses. Nobody is paying attention to ventilation. We still have a lot of overcrowding in informal settlements where rate of transmission is high.”* (A3).

### Health system challenges

Many KIs noted that the South African healthcare system suffers from many systemic challenges which cause TB to thrive. Understaffing in health facilities, mismanagement, lack of funds, noncompliance with basic infection control and poor TB surveillance were some of the health system challenges pointed out by the KIs. A government official who acknowledged poor infection control practices as a driver of the TB epidemic explained that:

“*when we do our inspections, we find gross noncompliance with basic infection control, basic things that are needed like PPE [personal protective equipment] and this is because the employer does not provide resources. There is no permanently appointed person looking after infection control in some of the institutions.*” (G7).

*Infection control is really lacking in clinics and hospitals. We need to take IC seriously* (N2).

“*There is a barrier of non-compliance from high up in healthcare.*” (G5)

Patient non-disclosure of TB status to healthcare workers was also cited by a KI. A healthcare worker who was once a victim of nosocomial TB transmission noted that:

*“the patients don’t always honestly tell us they have TB. I was working in a private dialysis unit. I knew of HIV status but not TB.*” (N1).

Resource allocation was frequently mentioned as a health system factor that impacts the healthcare system’s ability to combat TB. Many KIs explained that the public sector which serves the majority of the population is not adequately resourced. A KI noted that:

“*I don't believe the public system has been adequately funded.”* (N4).

*“Budget affect our department. Even before the financial year is done, we have exhausted the budget.”* (G4).

*“Budget is definitely one of the biggest issues affecting TB in South Africa.”* (G10).

*“There is political will to end TB but the issue is with resources.”* (G9)

Every KI noted that TB treatment was provided free of charge in South Africa. However, some KIs drew attention to the fact that other related costs such as transportation costs, limited access to treatment. Consequently, the efforts of the government to respond to the increased burden of TB were mitigated by the aforementioned challenge. A hospital head disagreed that transportation cost was a barrier as she explained that:

“*the only slight factor might be two things: transport might be one. But even that it shouldn’t be a problem because we offer transport to our patients to our facility and from our facility to the community. …. The other thing is the issue of the socioeconomic thing…you need to take the medicine with food and if there is no food then there is a problem.”* (H1).

An academic expert pointed out that “*in this country, there are no barriers [to TB treatment]”* (A2).

A representative of an NGO however emphasized that:

*“there are barriers to accessing treatment. We don’t bother to counsel patients for TB treatment. [Also] we do not have TB treatment in the private sector.”* (N3).

A KI further explained that infected people who have medical insurance are usually captured within the healthcare system but

“*those without medical aid especially are lost to tracing. Others simply don’t trust the care at the [public]clinics and would go to their own doctors [private].*” (G6).

A KI who had been cured of MDR TB cited the side effects of TB medications as a barrier to treatment adherence. The respondent explained:

*“It’s hard to take the medication; the side effects of the drug. You go through the day awful and you go to bed and can’t sleep. I couldn’t drive because of joint pain. The second reason is it’s a long-term treatment. Also, the drugs cause terrible depression. I was on treatment for 17 months.”* (N1).

The lack of investment in new drugs also came up in the interviews. A KI explained:

*“So, you have a major epidemic, you have essentially an old regimen which hasn’t changed in 50 years, you have pressure to produce resistance and the spread of resistance in communities.”* (G8).

A KI also mentioned the inadequate human resource for health in South Africa explaining that: “*the issue is overload. We don’t have enough healthcare workers to deal with the TB burden in the country.*” (G1).

### The HIV epidemic

The contribution of HIV as a driver of TB was widely stressed by the KIs. Almost every KI noted that the HIV epidemic reversed any medical or health system gains made in the management of TB, especially around the time when South Africa’s political system was improving (post-1994). The rates of co-infection of TB and HIV have been very high. A KI explained:

*“You could say that for the last two decades the high TB rates have been driven by HIV. Rates were still high prior to this period though.”* (A1).

Another KI explained that:

“*there was TB for a long time and then when the political system was changing or getting better, the HIV epidemic made it worse.*” (G6).

### Socio-cultural factors

TB infection is associated with many socio-cultural factors including masculinity, stigma, healthcare worker attitude and poor risk perception. Masculinity was blamed for driving TB among men. Per this account, men often delayed seeking care until their condition became very serious. A KI explained that the people at risk for TB are migrant workers, including those who work in the mines, agriculture, construction and other industries where men are the majority. A government official explained that*:*

*“it is just a masculinity issue. So, to the African man, the clinic is for the women and children. Men don’t go to the clinic.”* (G8).

The existence of stigma continues to heighten TB in South Africa. There is a profound stigma associated with TB because of its association with poverty and with HIV, itself highly stigmatized. KIs explained that this stigma prevents self-disclosure and care-seeking behaviour. A KI explained that:

*“[there is] still delay in patients seeking treatment. One of those reasons is there is still a stigma. No one wants to be diagnosed of TB. …. If you are employed, you don’t know how that might affect your employment and you don’t want to be unemployed.”* (H1).

A KI who had been cured of TB disease in the past recounted:

*“…the stigma…I had TB and acquired treatment, and no one knew about it because I was ashamed.*” (G2).

Underrating the risk of TB was also cited as a major determinant of the TB epidemic in South Africa. It was mentioned that people were not intensely concerned about the risks and impacts of TB as they were for other conditions such as HIV. A KI asserted that:

“*People are aware of HIV not TB. Half may know. Majority will care less. It has never been in the public domain.”* (A2).

Patients’ attitude towards the wearing of personal protective equipment (PPE) also increases the risk of infection. An academic expert explained that:

*“researchers, including myself at one point wanted to interview TB patients. A patient will not be willing to talk to me if I am wearing N95. They will think…is something wrong with me.”* (A3).

Consequently, healthcare workers commonly avoid the use of PPE, increasing their risk of acquiring TB.

An official also reiterated that although patients do not have to pay any fees at the clinic, some people prefer hospitals where marginal fees may be charged. It was explained that those patients believe the services in the clinics are sub-standard compared to those offered in hospitals.

“*They [the patients] don’t believe so much in clinics*” (G6), the official explained.

### Analysis of the strategic plan 2017–2022

Unlike many of the 22 high-burden TB countries that have not articulated clear strategic plans to combat TB, the current South African NSP aims to reduce TB incidence by at least 30%, reducing the incidence of TB from 834 cases per 100,000 population in 2015 to less than 584 cases per 100,000 by 2022. The NSP sets out eight goals accompanied by specific objectives and interventions for the achievement of these goals. A key feature of the NSP is a strong focus on HIV prevention among adolescent girls and young women. Table 2 contains the eight goals of the current NSP, corresponding focused areas of intervention, the scale of focus and the concurrent themes from the interview responses.

**Table 2:** Summary of the NSP 2017–2022 and concurrent themes from interviews responses

The goals of NSP 2017–2022	Focus areas of intervention	Scale of focus	Concurrent themes from interview responses
#1: Accelerate prevention in order to reduce new HIV and TB infections and new STIs. –Breaking the cycle of transmission	Biomedical preventive methods; sex education and environmental intervention for TB infection control	Micro, Meso, Macro	The HIV epidemic
#2: Reduce morbidity and mortality by providing treatment, care and adherence support for all. –Reaching 90–90-90 in every district	Treatment	Meso, Macro	Treatment related factors
#3: Reach all key and vulnerable populations with customised and targeted interventions. – Nobody left behind	Barriers of access to HIV, TB and STI treatment & prevention programmes by vulnerable populations	Macro	The mines and migrant labor system
#4: Address the social and structural drivers of HIV, TB and STIs, and link these efforts to the NDP-multi-department, multisector approach	Social and Structural drivers of HIV, TB and STIs	Meso, Macro	Colonization, political and economic system; infrastructural challenges
#5: Ground the response to HIV, TB and STIs in human rights principles and approaches. –Equal treatment and social justice	Human rights and stigma reduction	Meso, Macro	Socio-cultural factors
#6: Promote leadership at all levels and shared accountability for a sustainable response to HIV, TB and STIs. – Mutual accountability	Diverse leadership	Macro	NA
#7: Mobilise resources and maximise efficiencies to support the achievement of NSP goals and ensure a sustainable response. – Spend now to save later	Increased funding	Macro	Health system challenges
#8: Strengthen strategic information to drive progress towards achievement of NSP goals – Data-driven action	Monitoring and evaluation	Micro, Meso, Macro	NA

Of the eight goals set out in the NSP, the fourth goal specifically talks about the social and structural drivers of TB. According to the NSP, this “goal responds to the reality that the health of individuals is shaped by economic, social and environmental factors, such as poverty, gender discrimination, substance and alcohol use, and poor housing.” Specific objectives of this goal include: scaling up access to social protection for people at risk of and living with HIV and TB in priority districts, implementing economic development programmes for youth in priority areas, addressing barriers to prevention and treatment that arise from the design and construction of public housing etc. [17].

**Fig. 1:**
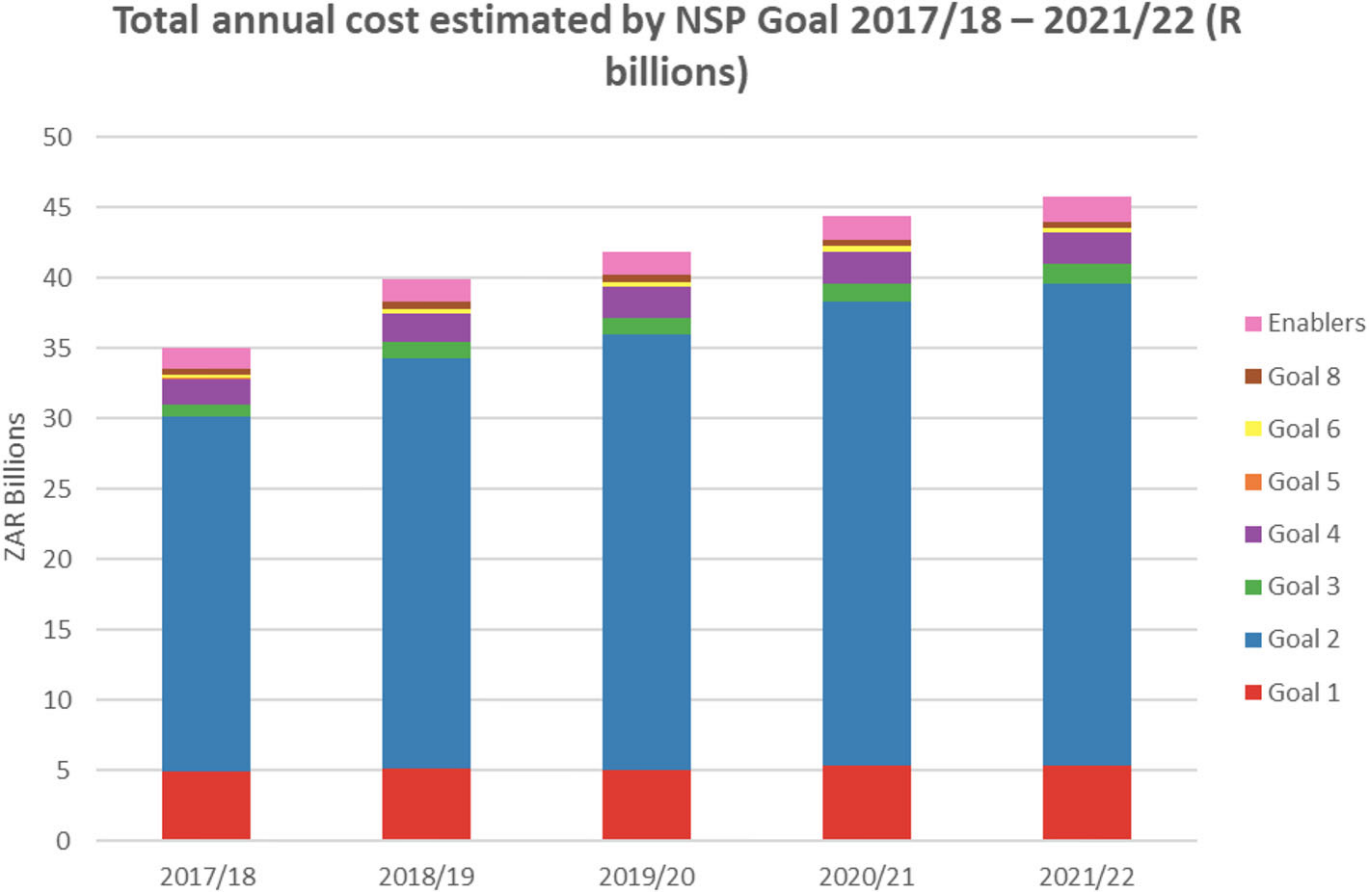
Total annual cost estimates by NSP Goal-2017/18–2021/22 (R billions). Adapted from “National Strategic Plan on HIV, STIs and TB 2017–2022” by South African National AIDS Council (SANAC), 2017

Despite a seemingly strong structural approach, the NSP’s projected cost estimates do not reflect this commitment. Of the eight goals, goal number four which specifically addresses the structural drivers has a significantly lower budget compared to goal number two, which contains the most curative elements (Fig. 1).

Apart from the eight goals, there are five critical enablers identified to strengthen systems to achieve the strategies set out in the NSP. These include 1) building strong social systems; 2) a focus on social and behaviour change communication; 3) effective integration of HIV, TB and STI interventions and services; 4) strengthening procurement and supply chain systems; and 5) strengthening human resources. The NSP identifies high impact TB interventions as those that produce social and behaviour change among healthcare professionals communicating important information to the community about the TB transmission [17].

Again the “enablers” component, which is supposed to provide systemic support for the achievement of the other goals, is still relatively deficient on budgetary allocation (Fig. 1).

## Discussion

This study focused on stakeholders’ perceptions of how upstream and systemic factors drive TB incidence, and how the South African NSP, the current policy instrument guiding TB prevention, management and control in the country, responds to these influential factors. Not surprisingly, there was strong concordance between the accounts of the KIs and the discourse in the NSP, spanning the political, socio-economic to health system and treatment-related factors.

Interviewees identified funding as a major issue in the fight against TB, noting that TB programs were heavily underfunded. There have been calls for increasing expenditures for this purpose; however, it can be observed that although the NSP appears to prioritize a structural or macro-level approach, its recommended cost apportionments show otherwise.

Many of the KIs expressed similar views regarding the origin of TB in South Africa. This consensus parallels the *virgin soil theory* which posits that TB was introduced by contact with Europeans [28, 29]. Some KIs recounted this historical event as important in the discussion around the drivers of the South African TB epidemic. Although considerable practical evidence supports this position which gained prominence in the early 1900s [30], one needs to be mindful of interests served by the propagation of this narrative. As Packard notes, this account has often been used as “…a means of deflecting attention from the appalling conditions under which Africans lived and worked” (Packard, [31], p.32). The poor living and working conditions were after all themselves largely driven by the same industrial expansion, particularly in the mining sector from the late 1800s to the early 1900s, that had driven the migration of Europeans.

The inextricable link between TB and mining which has received a lot of attention in the literature [32–34] was highlighted by the KIs of this study. There seemed to be considerable consensus that the mining industry is a significant driver of the epidemic. Indeed, only few TB experts would disagree with the mining industry’s enduring reputation of poor working conditions. Although KIs largely agreed that the risk of TB infection is high among miners, many also concurred that the mining companies provide better occupational health services, including TB treatment, to miners compared to what the general population receives, although social protection and health services for ex-miners and contract workers are often fraught with challenges [35, 36]. There is also a major Global Fund initiative underway to combat TB in this sector [37] and considerable other efforts [38], however attention to associated implications for broader community spread and disease control has been limited. While the efforts underway are certainly laudable, the fact that none of these are explicitly addressed in the Strategic Plan to combat this disease is problematic, as such failure to do so contributes to continued neglect of needed focus on underlying drivers.

Racial and economic inequality was a major theme from the interviews that is not adequately addressed in the NSP*.* As a social disease, TB is highly sensitive to structural violence [39]. With South Africa’s overall state of economic development, the country should not be burdened by TB at this rate. However, due to its massive income and health-related inequalities [40, 41], the disease continues to persist. Income inequality has worsened over the years with South Africa’s Gini coefficient (a measure of inequality) increasing from 0.59 in 1993 to 0.63 in 2011. About half of the population (53.8%) lives below the national poverty line [42]. In addition, the chronically underfunded public sector which serves about 84% of the population, is staffed by only 30% of doctors in the country [43]. In fact, this persistent structural violence [39] might explain South Africa’s poor health outcomes in the post-Apartheid era, despite considerable evidence of economic growth [43, 44]. It was agreed that TB largely affects the poor and/or those marginalized in society, a sub-population that also receives a lot of attention in the NSP. Interestingly, the point about socioeconomic status and the mining sector accounting for TB is weakened by the case of the Western Cape Province. The Western Cape Province is a relatively well-off province with comparatively better health services, yet this province has one of the highest TB rates in the country even in the pre-HIV era. It should also be noted that there are no mines in this region, assuming the hypothesis that mining drives TB [45] was valid. Reasons for this paradox are inconclusive; they are speculative at best. One hypothesis is the high rate of migration of foreign farm workers from other regions to this area. A more plausible explanation for this could be the huge economic inequality in this area despite its overall wealth. It is important that future NSPs aim to address this inequality, not only in this region but in the country as a whole.

One key goal of the World Health Organization’s End TB strategy is a zero catastrophic cost for patients and families affected by TB [46]. This shows that the socioeconomic needs of those patients and their families need to be appropriately addressed in order to reduce the high rates of infections. One of the socio-economic barriers is access and affordability of transportation to get to the facilities; some patients report late at health facilities because they do not have the money for transportation. Although some facilities provide transportation for patients, as articulated by a hospital head, this is still a major barrier for accessing TB care for many and better understanding is needed of the barriers that patients face. Even though a comprehensive view of all the barriers that prevent access to TB treatment is lacking, it is well-known that socioeconomic deprivation, poor housing, overcrowding, and malnutrition continue to drive the epidemic in South Africa. A strategic plan to tackle TB needs to allocate funding directly to these factors.

If the battle against TB is to be won, the many challenges confronting the South African health sector need to be tackled. Currently, treatment for TB is not offered by the private sector; it was suggested that treatment be extended to the private sector to extend access.

The impact of stigma on TB-related care was well highlighted in the results of this study and confirmed the findings of several studies [47–49]. As noted by an official, the problem however is the difficulty in measuring this construct accurately to know the extent to which stigma on TB-related care is a barrier.

The study of key informants’ perceptions revealed inadvertently that there was no consideration of lost revenues as a relevant upstream pathway that contributed to the chronic underfunding of TB services. Illicit financial flows (IFFs), the unrecorded movement of capital out of a country in contravention of the regulations of that country and various accounting practices, for example, have been noted as contributing to the weakening of the health systems of many African countries [50]. This suggests that awareness of IFFs and their consequences is low among experts in the TB-OHS community. This absence indicates that further examination of this matter is warranted.

While this study offers significant contributions to the literature on perceptions by decision-makers and TB experts regarding the structural drivers of diseases, it is not without limitations. A major limitation is that, although data saturation was reached with the sample size of twenty KIs, this study could have benefitted from a more diverse group of KIs who would potentially offer other perspectives. Many of the KIs were recruited through networks of public health and occupational health researchers. This might have influenced the results in the sense that it is possible to assume a disproportionate occupational health bias in the findings. Future studies could include other stakeholders such as infection control practitioners, human resource managers, labour unions and representatives from joint health and safety committees of hospitals, as well as a more diverse array of clinical managers at various levels and from other government departments beyond health. Further research is needed to elucidate these other perspectives. Also, the reliance on perceptions speaks to a need and opportunity for clearer identification of constructs that could be more directly and systematically monitored.

It could be argued that the budgetary allocation specified was low, as structural issues related to social determinants of health are covered in strategic plans in other departments or related to other issues. Nonetheless, we argue that strategic plans to combat TB in high burden settings need to more directly consider such drivers to prompt the necessary changes and reduce the burden of this and other such diseases.

## Conclusion

Despite some progress, TB mortality is still very high in South Africa. Strategies that focus solely on effective case management of TB have not yielded the desired results of reducing TB’s burden of disease in South Africa. While it is understandable that investment in newer treatment and technologies continues to receive attention as a way to respond to a disease that is present in epidemic proportions, it is somewhat disconcerting that the funding allotment to address macro-level drivers of TB remains neglected and disassociated with achieving specific strategic targets. Indeed, the healthcare system has been unable to control the epidemic through the medical treatment model alone. There appears to be a shift in direction per the current TB policy prescriptions as it appears to commit to addressing the structural drivers; however, the projected budgetary allocations towards addressing these specific drivers do not reflect this level of commitment. Thus, funding allocation and other tangible commitments need to be pursued especially in areas related to poverty reduction. Funding should also more broadly address the other macro-level issues identified in this study. The findings of this study further suggest the need to rethink current approaches and incorporate interventions that address upstream processes contributing to the persistence of this preventable and curable disease. These structural interventions should be implemented simultaneously with interventions that are committed to strengthening diagnosis and treatment of TB; these combined efforts will continue to reduce the burden of TB.

## Data Availability

The datasets used and analyzed during the current study are available from the corresponding author on reasonable request.

## Abbreviations

XDR: Extremely drug resistant
HCW: Health care workers
HIV: Human Immunodeficiency Virus
IFF: Illicit financial flow
KI: key informants
MDR: Multi-drug resistant
NSP: National Strategic Plan
PPE: personal protective equipment
STIs: Sexually Transmitted Infections
TB: Tuberculosis
WHO: World Health Organization
